# HTLV-I proviral load in Argentinean subjects with indeterminate western blot patterns

**DOI:** 10.1186/1742-4690-8-S1-A245

**Published:** 2011-06-06

**Authors:** Andrea Mangano, Natalia Altamirano, Mirta Remesar, María B Bouzas, Paula Aulicino, Inés Zapiola, Ana D Pozo, Luisa Sen

**Affiliations:** 1 Laboratorio de Biología Celular y Retrovirus-CONICET, Hospital “J. P. Garrahan”, Ciudad Autónoma de Buenos Aires, Argentina; 2 Servicio de Hemoterapia, Hospital “J. P. Garrahan”, Ciudad Autónoma de Buenos Aires, Argentina; 3 Unidad de Virología, Hospital Muñiz, Ciudad Autónoma de Buenos Aires, Argentina

## Background

A considerable high proportion of HTLV-I/II seroindeterminate blood donors have been documented in many countries including Argentina. In 5-10% of indeterminate Western Blot (WB) cases proviral sequences are detected. The aim of the study was to evaluate HTLV-I proviral load (pVL) in WB indeterminate PCR positive cases.

## Methodology

A total of 87 indeterminate WB samples (HTLV blot 2.4 assay- Genelabs Diagnostics-) were studied over a 10 year period referred from the Blood Bank at Garrahan Hospital (n=83) and from the Virology Unit at Muñiz Hospital (n=4). HTLV-I and –II tax and pol proviral sequences were amplified by in-house nested PCR assays. HTLV-I pVL was estimated by a quantitative real-time PCR assay targeting the HTLV-I pol gene and the albumin gene as normalizer. The limit of detection of the assay was 2.6 log10 copies of HTLV-I/106 PBMCs (0.04% copies/100 PBMCs).

## Results

In 8/87 samples HTLV proviral sequences were amplified, 7 HTLV-I and one HTLV-II. Proviral load was detectable in 4 of the 7 HTLV-I positive samples ranging from 2.72 log10 copies/106 PBMCs (0.05%) to 4.65 log10 copies/106 PBMCs (4.5%), but was undetectable in the remaining 3 (<0.04%). One of the subjects followed for 5 years remained with low pVL (median = 0.36%, range 0.05-1.43%) and without changes in the WB profile.

## Conclusions

Detectable HTLV-I proviral load in half of the cases with indeterminate WB profiles with positive PCR underlies the importance to conduct follow-up studies to evaluate the evolution of the infection.

